# CircRNA has_circ_0069313 induced OSCC immunity escape by miR-325-3p-Foxp3 axes in both OSCC cells and Treg cells

**DOI:** 10.18632/aging.204068

**Published:** 2022-05-16

**Authors:** Yiyang Chen, Zeyu Li, Jianfeng Liang, Jiayu Liu, Jiansuo Hao, Quan Wan, Jiameng Liu, Chongdai Luo, Zhiyuan Lu

**Affiliations:** 1 Department of Oral and Maxillofacial Surgery, Stomatology Medical Center, Guangzhou Women and Children’s Medical Center, Guangzhou Medical University, Guangzhou, China; 2 School of Stomatology of Qingdao University, Qingdao, China; 3 Department of Oral and Maxillofacial Surgery, Hospital of Stomatology, Sun Yat-Sen University, Guangzhou, China

**Keywords:** circRNA, OSCC, cancer immunity, PDL1, Treg

## Abstract

Introduction: CircRNAs are engaged in the tumorigenesis and progression of oral squamous cancer cells (OSCC). However, the function and underlying mechanism of circRNAs on tumor-associated immunity escape are largely unknown.

Materials and methods: We analyzed the expression pattern of has_circ_0069313 in our in-house database and its correlation with OSCC prognosis. Immunohistochemistry was applied to detected PDL1 expression. RNA fluorescence *in situ* hybridization was applied to detect subcellular location of circRNA. A luciferase activity assay was used to detect the interaction of has_circ_0069313 and miR-325-3p and its downstream target, Foxp3. Exosomes were collected to detect the exosomal circRNAs and co-culture assays were performed to detect the function of exosomal circRNAs on Tregs.

Results: has_circ_0069313 was upregulated in OSCC tissues and predicts poor prognosis. has_circ_0069313 promotes immunity escape through inhibiting miR-325-3p-induced Foxp3 degradation. has_circ_0069313 is an exosomal circRNA and the transfer of has_circ_0069313 to Treg cells promotes the Treg function through maintaining Foxp3 levels.

Conclusion: Our results indicate that has_circ_0069313 induces OSCC immunity escape via the miR-325-3p-Foxp3 axis in both OSCC cells and Treg cells.

## INTRODUCTION

Oral cancer is the sixth most frequent malignancy worldwide and the oral squamous cell cancer (OSCC) subtype accounts for 90% of cases [1]. At present, therapy is only effective for early-stage OSCC patients while the reported 5-year overall survival of such patients is less than 50% [2]. Various studies have shown that excessive alcohol consumption, smoking and HPV infection are the main associated risk factors contributing to the development of OSCC [3]. The current therapies targeting OSCC are still far from satisfactory, therefore there is an urgent need to predict new biomarkers and therapeutic targets for OSCC patients [4].

Circular RNAs are generally reported as non-coding RNAs which play vital roles in the progression of physiology and pathology [5, 6]. CircRNAs mostly function as competing endogenous RNAs (ceRNAs) and RNA binding proteins (RBPs) partners. For example, circular RNA circSDHC serves as a sponge for miR-127-3p to promote aggressive phenotypes of renal cell carcinoma through CDKN3/E2F1 pathway, and circNDUFB2 inhibits non-small cell lung cancer growth and proliferation by targeting IGF2BPs [7, 8]. Nevertheless, the potential function and underlying molecular mechanism of circRNAs on anti-tumor immunity in OSCC remain still largely unknown.

In the present study, we analyzed the expression level of has_circ_0069313 in OSCC patients and reveal its’ biological functions and potential mechanism in OSCC cell lines. Our results showed that for the first time has_circ_0069313 promotes the tumor immunity escape and suggested it as the potential therapeutic target.

## RESULTS

### has_circ_0069313 is upregulated and correlate with poor prognosis in human OSCC

**Figure 1:**
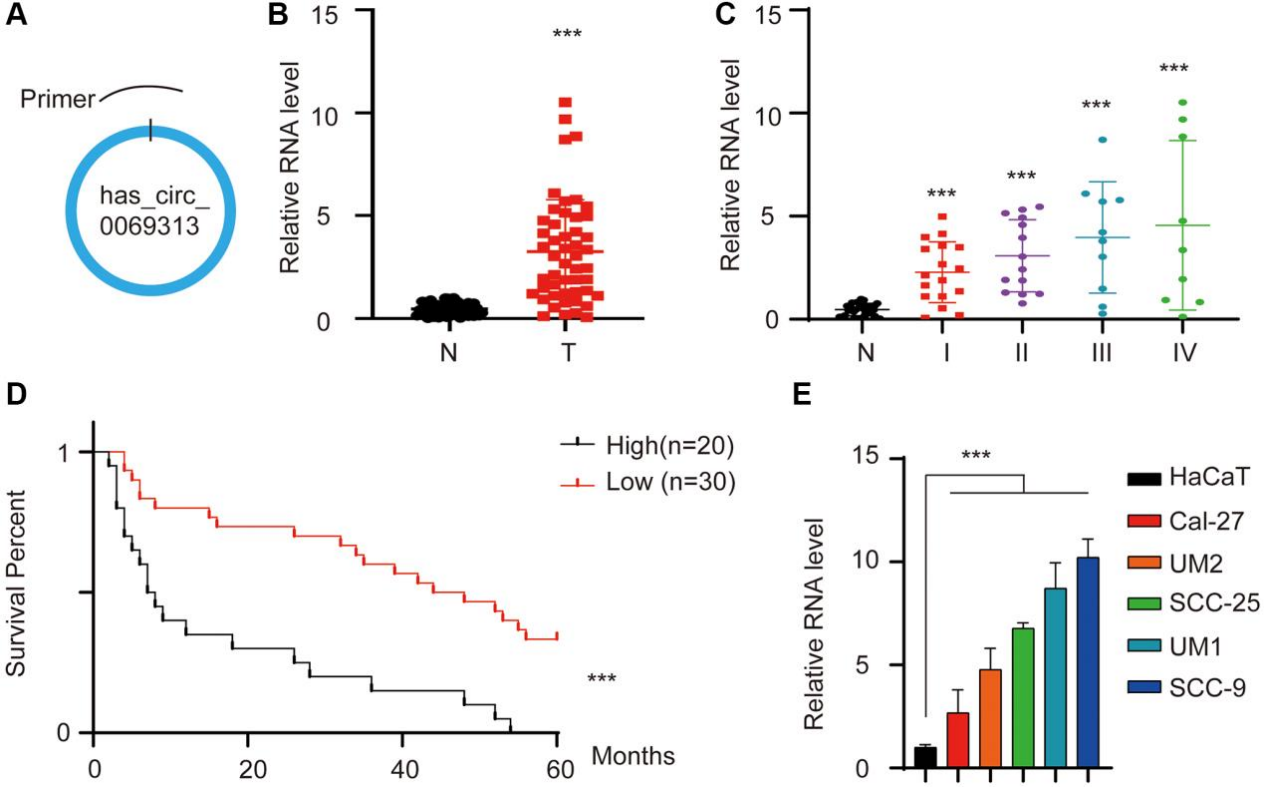
**has_circ_0069313 was upregulated in OSCC and predicts poor prognosis.** (**A**) The graphic illustration of has_circ_0069313, the specific primer was designed to target the junction. (**B**) The relative level of has_circ_0069313 in our in-house database. 50 paired OSCC and normal tissues were collected and subjected to qRT-PCR, the RNA level was detected using junction-specific primers. (Student’s two-tailed paired test, ^***^p < 0.001). (**C**) The relative level of has_circ_0069313 in patients with different TNM stages in our in-house database. The 50 OSCCs were divided into 4 TNM stages and the RNA level was detected and normalized. (Student’s two-tailed paired test, ^***^p < 0.001). (**D**) The overall survival analysis of the 50 OSCC patients. Patients were divided into two groups with the mean level of has_circ_0069313 as the cutoff. The overall survival analysis was applied. (**E**) The relative has_circ_0069313 level in OSCC cell lines and normal epithelial cells.

To detect the expression pattern of has_circ_0069313 in OSCC patients, we designed the junction-specific primers shown in Figure 1A and analyzed the relative level of has_circ_0069313 in our in-house database. The results indicated that has_circ_0069313 was upregulated in tumors and was correlated with the stage of the disease. Developed tumors harbor higher levels of has_circ_0069313 (Figure 1B, 1C, ^***^p < 0.001). We next divided the whole cohort into two groups, ‘high has_circ_0069313’ and ‘low has_circ_0069313’, taking the mean level as the cut-off. Patients with has_circ_0069313 higher than the mean value were identified as ‘high’, otherwise as ‘low’. Overall survival analysis was applied. Patients with high levels of has_circ_0069313 have shorter middle survival time (Figure 1D, ^***^p < 0.001). We next detected has_circ_0069313 levels in OSCC cell lines and normal epithelium cells. has_circ_0069313 was higher in OSCC cells compared with normal cells (Figure 1E, ^***^p < 0.001).

### has_circ_0069313 was associated with CD8+ T cell infiltration

**Figure 2:**
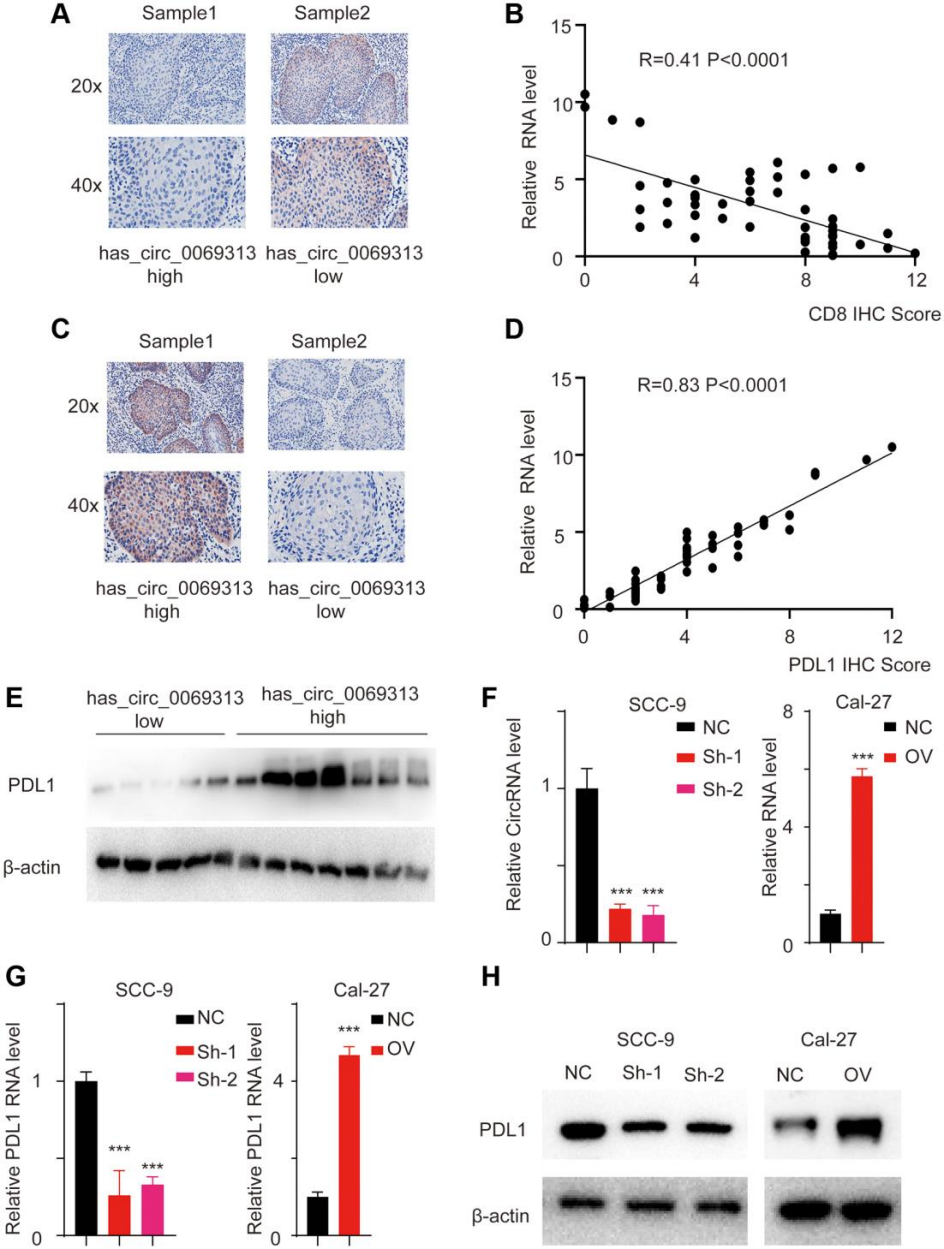
**has_circ_0069313 correlated with PDL1 level in OSCC samples.** (**A**) Samples were collected and subjected to IHC using the CD8 antibody. The representative images were shown with a 200× and 400× magnification. (**B**) The relative has_circ_0069313 level and CD8 IHC score were collected and regression analysis was applied, R = 0.41, *p* < 0.001. (**C**) Samples were collected and subjected to IHC using the PDL1 antibody. The representative images were shown with a 200× and 400× magnification. (**D**) The relative has_circ_0069313 level and PDL1 IHC score were collected and regression analysis was applied, R = 0.83, *p* < 0.001. (**E**) Samples were collected and subjected to immunoblot, PDL1 was detected as indicated. (**F**) has_circ_0069313 was knocked down using junction specific shRNAs and overexpression cell was established using the OV plasmid. has_circ_0069313 was detected and normalized. (Student’s two-tailed paired test, ^***^p < 0.001). (**G**) The relative PDL1 RNA level in cells with indicated modifications. (**H**) Immunoblot of PDL1 in cells with indicated modifications.

We collected has_circ_0069313 high and has_circ_0069313 low samples and subjected them to immunohistochemistry (IHC) assay. Staining with CD8 antibody was used to detect the effector T cell infiltration. The representative images were shown in Figure 2A and the correlation between has_circ_0069313 RNA level and CD8 IHC score was analyzed in Figure 2B. The results indicated that has_circ_0069313 was negatively correlated with the CD8 IHC score, indicating that CD8 effector T cells were less infiltrative in high has_circ_0069313 patients. PDL1 (programmed death-ligand 1) is a vital immunity checkpoint and is responsible for the CD8 effector T cell inhibition. We thus detect the PDL1 expression pattern in patient samples. IHC assay and regression analysis was applied as required. The representative image is shown in Figure 2C and regression analysis was applied in Figure 2D. Higher has_circ_0069313 was correlated with higher PDL1 levels. This finding was also confirmed using immunoblot assays (Figure 2E). We next established a stable knockdown cell line using lentivirus with junction-specific shRNAs and an overexpression cell line using the OV plasmid with has_circ_0069313 cDNA (see Methods). The relative levels of has_circ_0069313 and PDL1 were detected, showing that in the has_circ_0069313 knockdown cell line the expression of PDL1 decreased while PDL1 increased in has_circ_0069313 overexpressing cells (Figure 2F–2H).

### has_circ_0069313 directly binds with miR-325-3p

**Figure 3:**
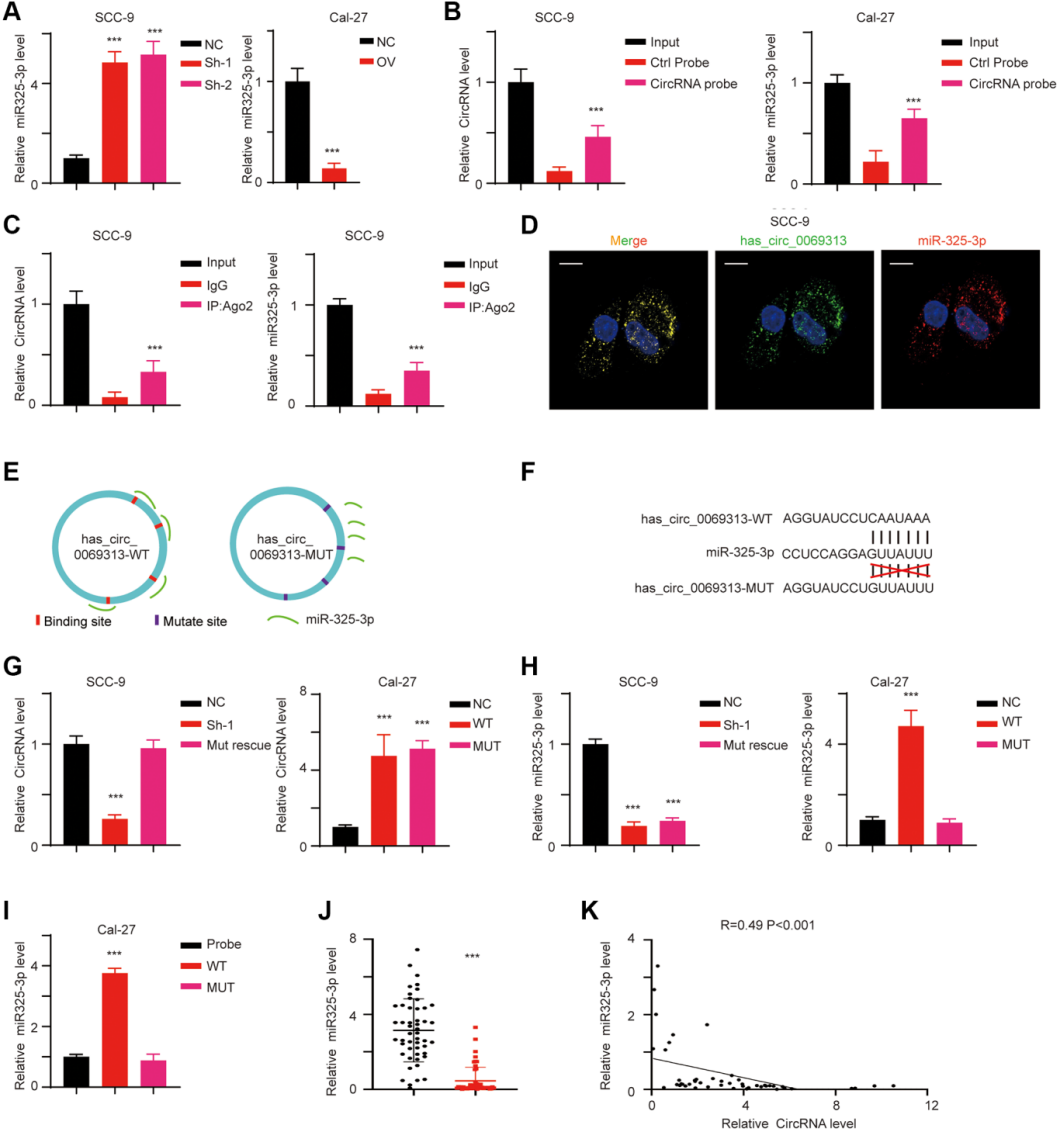
**has_circ_0069313 directly targeted miR-325-3p.** (**A**) The relative RNA level of miR-325-3p of indicated cells. (Student’s two-tailed paired test, ^***^p < 0.001). (**B**) RNA probe was used in RNA pull-down assay and the circRNA level and miRNA level were detected (Student’s two-tailed paired test, ^***^p < 0.001). (**C**) RIP assay was applied using Ago2 antibody. The relative has_circ_0069313 and miR-325-3p were detected and normalized (Student’s two-tailed paired test, ^***^p < 0.001). (**D**) FISH using the junction specific probe and miR-325-3p probes, scale 20 μm. (**E**) The graphic illustration of has_circ_0069313 WT and MUT establishing strategy. (**F**) The detailed Mut allele establishment strategy. (**G**) SCC-9 sh-1 cells were transfected with the has_circ_0069313 Mut allele and named as Mut rescue. Cal-27 was transfected with the has_circ_0069313 Mut allele and was named as MUT. Cells were subjected to qRT-PCR and has_circ_0069313 was detected and normalized (Student’s two-tailed paired test, ^***^p < 0.001). (**H**) miR-325-3p was detected and normalized in cells with indicated modifications (Student’s two-tailed paired test, ^***^p < 0.001). (**I**) RNA pull-down assay was applied using junction-specific primers and the complex was subjected to qRT-PCR and miR-325-3p was detected (Student’s two-tailed paired test, ^***^p < 0.001). (**J**) The relative miR-325-3p level in our in-house database (Student’s two-tailed paired test, ^***^p < 0.001). (**K**) The regression analysis between has_circ_0069313 and miR-325-3p in our in-house database (R = 0.49, *p* < 0.001).

CircRNAs exert their function mostly through acting as competing endogenous RNAs. After searching CircNET, RNAhybrid, and miRanda tools and we identified miR-325-3p as the candidate target miRNA. We first detected the expression of miR-325-3p in stable cell lines, and results indicated that miR-325-3p is upregulated in has_circ_0069313 knockdown cell lines but decreased in has_circ_0069313 overexpression cells (Figure 3A, ^***^p < 0.001). We next applied the RNA pull-down assay using the circRNA specific junction probe and found that miR-325-3p was detectable in the complex (Figure 3B, ^***^p < 0.001). As the interaction between circRNAs and miRNAs is carried with the help of Ago2, we applied RNA immunoprecipitation (RIP) assay using Ago2 and has_circ_0069313 and miR-325-3p were detected. The results showed that has_circ_0069313 and miR-325-3p were both detectable in the RIP complex (Figure 3C, ^***^p < 0.001). Fluorescence *in situ* hybridization (FISH) was applied using the junction-specific probe and a miR-325-3p probe. The results also confirmed the interaction (Figure 3D, ^***^p < 0.001). For further evidence, we thus established a has_circ_0069313 WT and a MUT allele (Figure 3E). The interaction residues were shown in Figure 3F and the MUT allele was established by replacing A to U, U to A, C to G, G to C, respectively. We next transfected the MUT allele into the has_circ_0069313 knocked down cell line and named it ‘Mut rescue’. The relative has_circ_0069313 and miR-325-3p levels were detected as well. Results showed that in the Mut rescue cell line, the level of has_circ_0069313 was restored but the level of miR-325-3p was barely changed compared to the knockdown cells. Overexpression of Mut has_circ_0069313 failed to increase the level of miR-325-3p (Figure 3G, 3H, ^***^p < 0.001). RNA pull-down also confirmed this result (Figure 3I, ^***^p < 0.001). We next detected the relative RNA level of miR-325-3p and analyzed the correlation between has_circ_0069313 and miR-325-3p. The results showed that miR-325-3p was downregulated in OSCC samples and was in a negative correlation with has_circ_0069313 (Figure 3J, 3K, ^***^p < 0.001).

### has_circ_0069313 inhibits miR-325-3p mediated Foxp3 degradation

**Figure 4:**
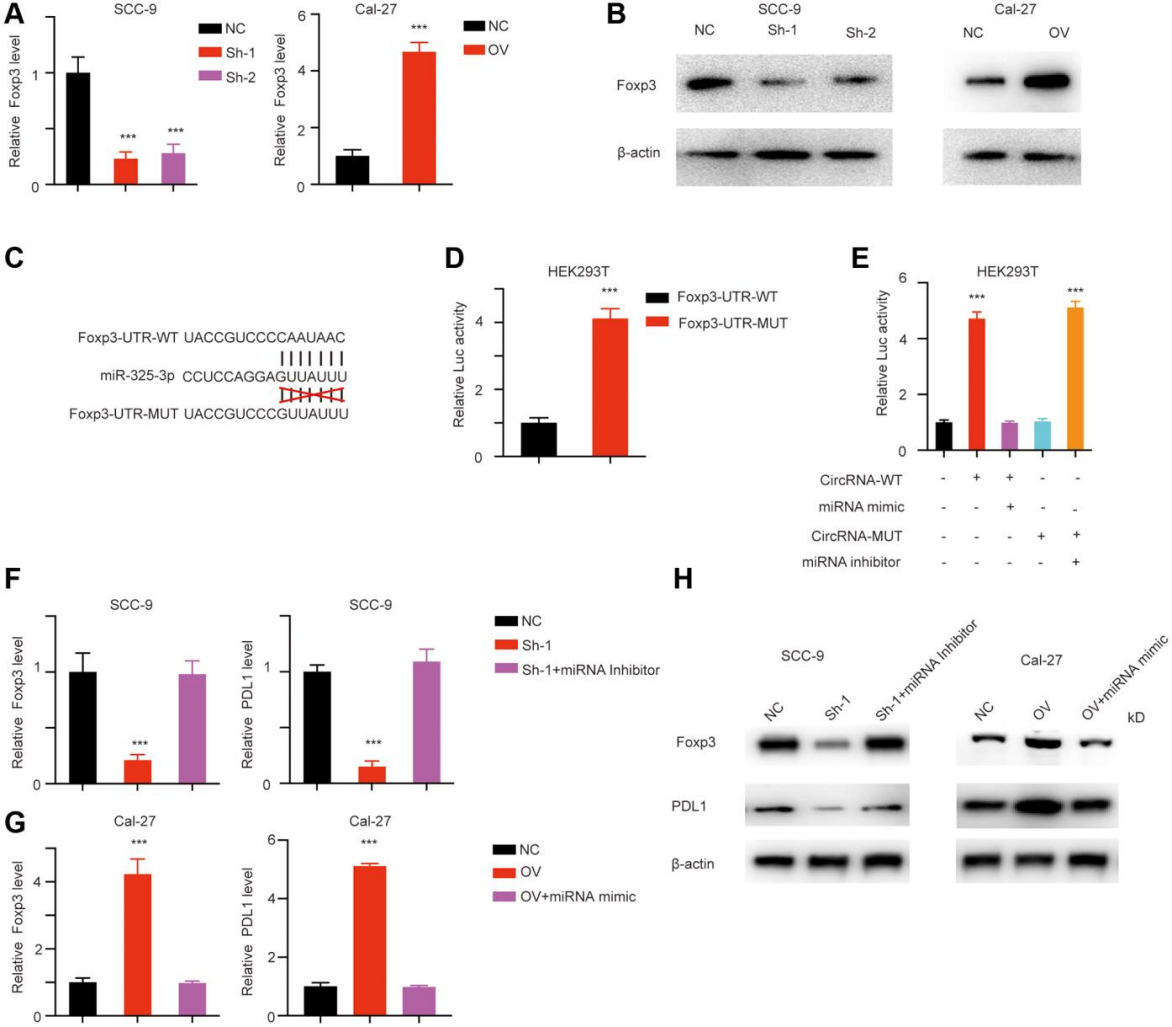
**has_circ_0069313 inhibited miR-325-3p induced foxp3 degradation.** (**A**) The relative Foxp3 level in cells with indicated modifications (Student’s two-tailed paired test, ^***^p < 0.001). (**B**) Immunoblot was applied to detect Foxp3 in cells with indicated modifications. (**C**) The detailed luciferase activity establishing strategy. (**D**) The luciferase activity reporting plasmid was transfected into HEK293T cells. The luciferase activity was measured and normalized (Student’s two-tailed paired test, ^***^p < 0.001). (**E**) HEK293T was transfected with has_circ_0069313, has_circ_0069313 MUT, miR-325-3p mimic, miR-325-3p inhibitor alone or in combination. Luciferase activity was detected and normalized (Student’s two-tailed paired test, ^***^p < 0.001). (**F**) SCC-9 cells were transfected with shRNA and shRNA plus miRNA inhibitor, relative Foxp3 level was detected and normalized (Student’s two-tailed paired test, ^***^p < 0.001). (**G**) Cal-27 cells were transfected with OV plasmid, OV plasmid plus miRNA mimic, the relative Foxp3 and PDL1 level was detected and normalized (Student’s two-tailed paired test, ^***^p < 0.001). (**H**) Immunoblot was applied to detect PDL1 and Foxp3 in cells with indicated modifications.

miRNAs prefer to bind to the untranslated region of target genes mRNA and promote its degradation and eventually the decrease of the target genes levels. Using Targetscan we identified Foxp3 as the potential downstream target of miR-325-3p. We next detected the RNA level and protein level of Foxp3 in different cell lines. Foxp3 decreased in has_circ_0069313 knockdown cells but increased in has_circ_0069313 overexpressing cells (Figure 4A, 4B, ^***^p < 0.001). We established Foxp3 WT and MUT luciferase activity reporter assays and transfected them into OSCC cell lines and the relative luciferase activity was detected (Figure 4C, ^***^p < 0.001). The results indicated that the Foxp3 luciferase increased with the MUT allele (Figure 4D, ^***^p < 0.001). We then transfected with has_circ_0069313, WT/MUT miR-325-3p mimic/inhibitor alone or in combination, relative luciferase activity was detected and the results suggested that has_circ_0069313 inhibited miR-325-3p induced Foxp3 degradation through binding to the 3′UTR of Foxp3 (Figure 4E, ^***^p < 0.001). We next transfected a miR-325-3p inhibitor into has_circ_0069313 knockdown cells and a miR-325-3p mimic in has_circ_0069313 overexpressing cells. Foxp3 and PDL1 were detected. The results indicated that Foxp3 and PDL1 levels were completely restored with the introduction of the inhibitor/mimic (Figure 4F–4H, ^***^p < 0.001).

### has_circ_0069313 is upregulated in OSCC derived exosome

**Figure 5:**
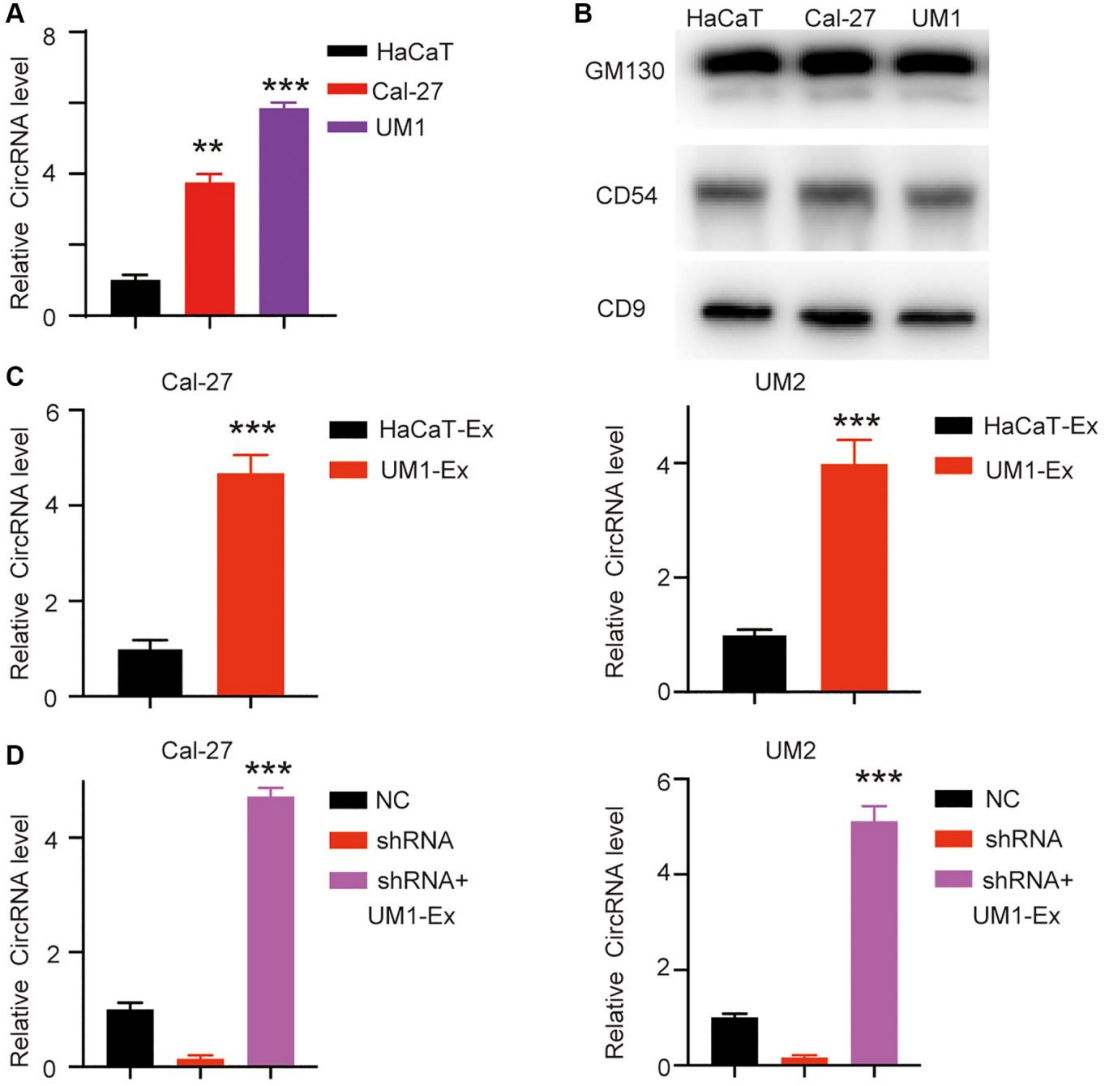
**has_circ_0069313 was detectable in exosome.** (**A**) Different exosomes were derived from different OSCC, the exosomes were then subjected to qRT-PCR and the relative has_circ_0069313 was detected (Student’s two-tailed paired test, ^**^p < 0.01, ^***^p < 0.001). (**B**) Immunoblot of exosome markers was detected. (**C**) Cal-27 and UM-2 were treated with UM1 derived exosomes and has_circ_0069313 was detected and normalized (Student’s two-tailed paired test, ^***^p < 0.001). (**D**) Cal-27 and UM-2 were transfected with shRNAs and then treated with UM1 derived exosomes. The relative has_circ_0069313 level was detected (Student’s two-tailed paired test, ^***^p < 0.001).

CircRNAs were reported to exert paracrine functions through exosomes [9]. We next collected the OSCC derived exosomes and detected the relative has_circ_0069313 level (Figure 5A, ^***^p < 0.001). Exosomes markers CD9, CD54, and GM130 were detected as loading controls [10] (Figure 5B). We next treated OSCC cells with tumor exosomes and detected the relative has_circ_0069313 level, and the results indicated that the intracellular has_circ_0069313 level increased after the tumor exosome treatment (Figure 5C, ^***^p < 0.001). We next transfected has_circ_0069313 specific shRNA into the cells and then treated with tumor exosome. The intracellular has_circ_0069313 was detected and shows an increased level as well (Figure 5D, ^***^p < 0.001). This result excluded the influence of epigenetic regulation and attributed the increase of the intracellular has_circ_0069313 to the exosome has_circ_0069313.

### Exosome has_circ_0069313 promotes Treg function through targeting Foxp3

**Figure 6:**
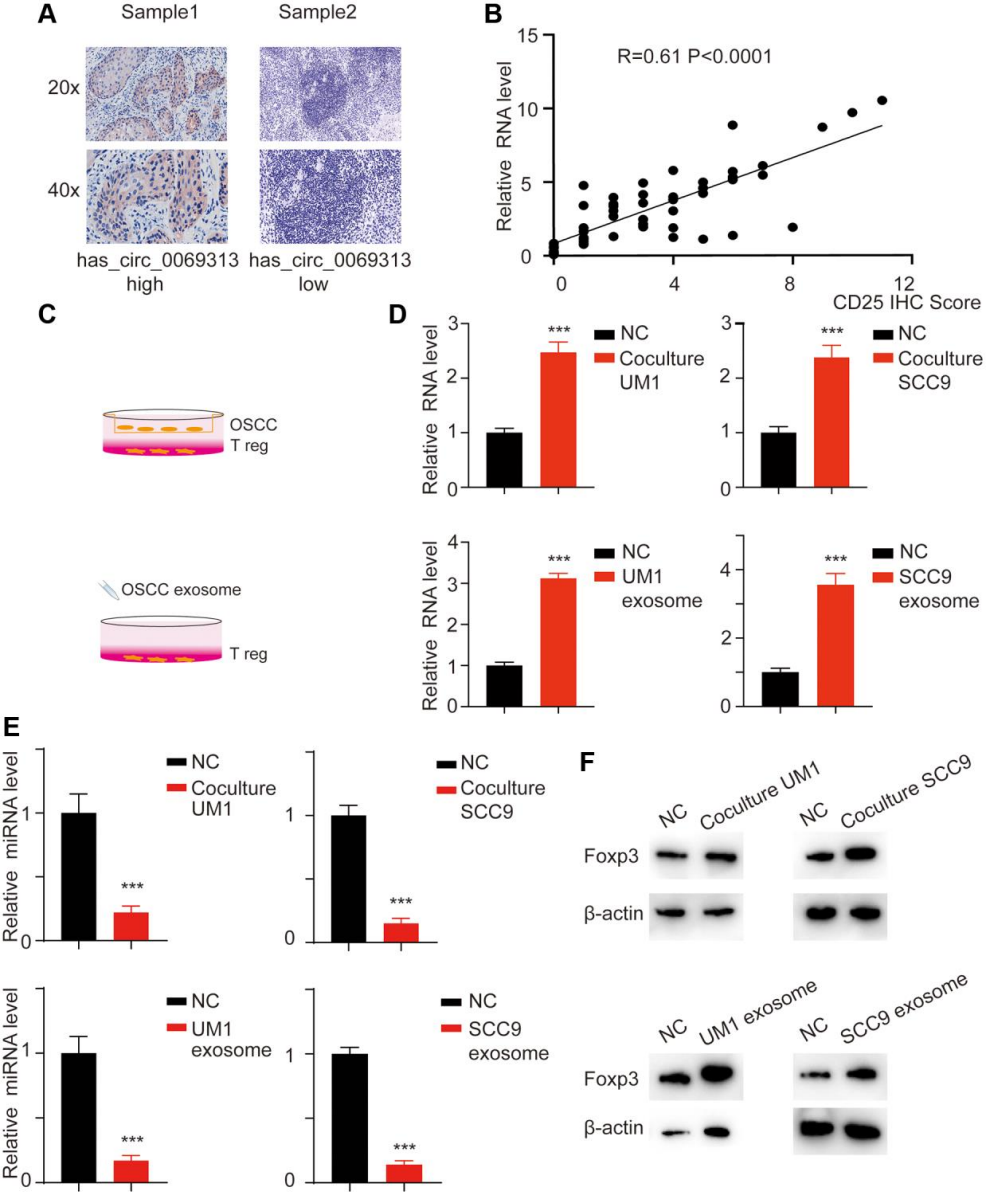
**has_circ_0069313 correlated with Treg infiltration.** (**A**) Samples were collected and subjected to IHC using the CD25 antibody. The representative images were shown with a 200× and 400× magnification. (**B**) The relative has_circ_0069313 level and CD25 IHC score were collected and regression analysis was applied, R = 0.61, *p* < 0.001. (**C**) The graphic illustration of coculture strategy and exosome treatment strategy. (**D**) Treg cells were treated with OSCC derived exosomes or co-cultured with OSCC. The Treg cells were collected and subjected to qRT-PCR, relative has_circ_0069313 was measured (Student’s two-tailed paired test, ^***^p < 0.001). (**E**) Relative miR-325-3p was detected in Treg cells with indicated treatments (Student’s two-tailed paired test, ^***^p < 0.001). (**F**) Immunoblot was applied for Foxp3 in Treg cells with indicated treatments.

We applied the IHC assay using the Treg cell marker CD25 and analyzed the correlation with has_circ_0069313. The results showed that has_circ_0069313 negatively correlates with CD25 (Figure 6A, 6B, ^***^p < 0.001). We thus hypothesized that OSCC has_circ_0069313 levels were associated with Treg function. We have previously indicated that has_circ_0069313 may exert its activity via a paracrine function. We next co-culture OSCC cells with Treg and treated Treg cells with OSCC derived exosomes (Figure 6C). has_circ_0069313 and PDL1 were detected. has_circ_0069313 and PDL1 increased when Treg cells were treated with OSCC derived exosomes (Figure 6D–6F, ^***^p < 0.001).

### Exosome has_circ_0069313 promotes the progression of OSCC *in vivo*

**Figure 7:**
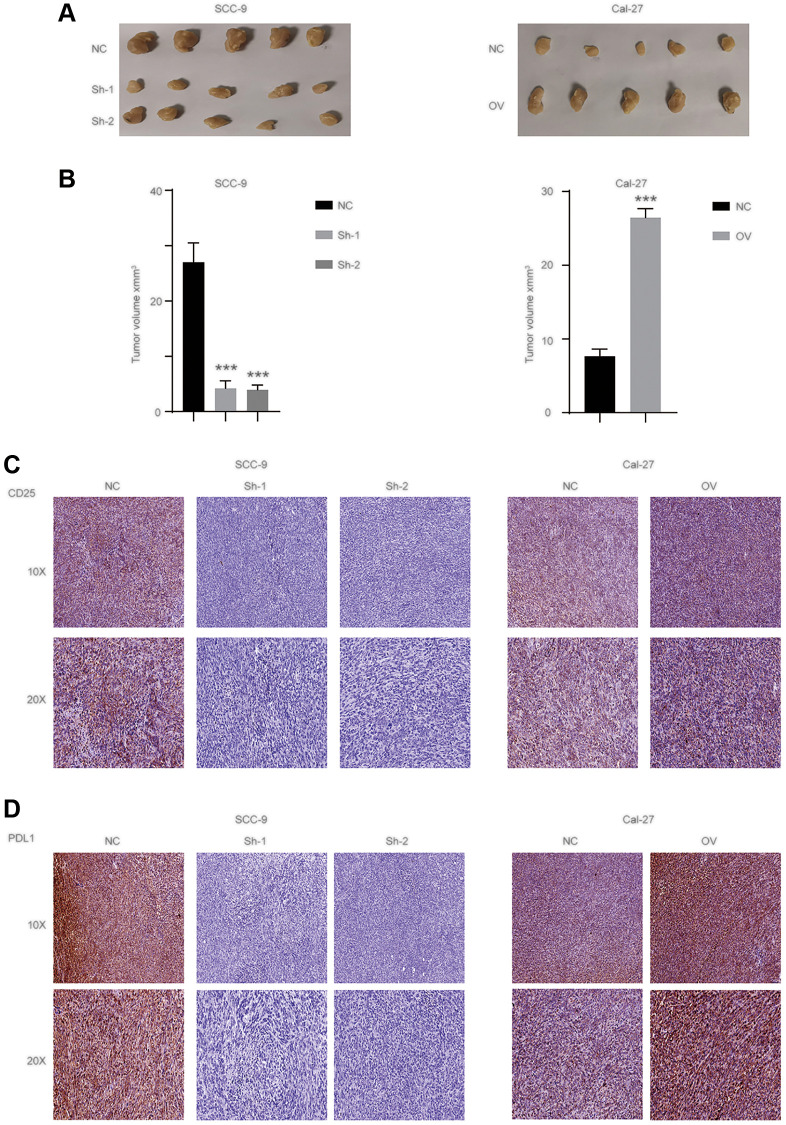
**has_circ_0069313 promoted the OSCC progression *in vivo*.** (**A**) The representative image of subcutaneous xenografts (*n* = 5 per group), scale 1 cm. (**B**) Tumors were collected and the volume was measured as indicated (Student’s two-tailed paired test, ^***^p < 0.001). (**C**) Samples were collected and subjected to IHC using the CD25 antibody. The representative images were shown with a 100× and 200× magnification. (**D**) Samples were collected and subjected to IHC using the PDL1 antibody. The representative images were shown with a 100× and 200× magnification.

**Figure 8:**
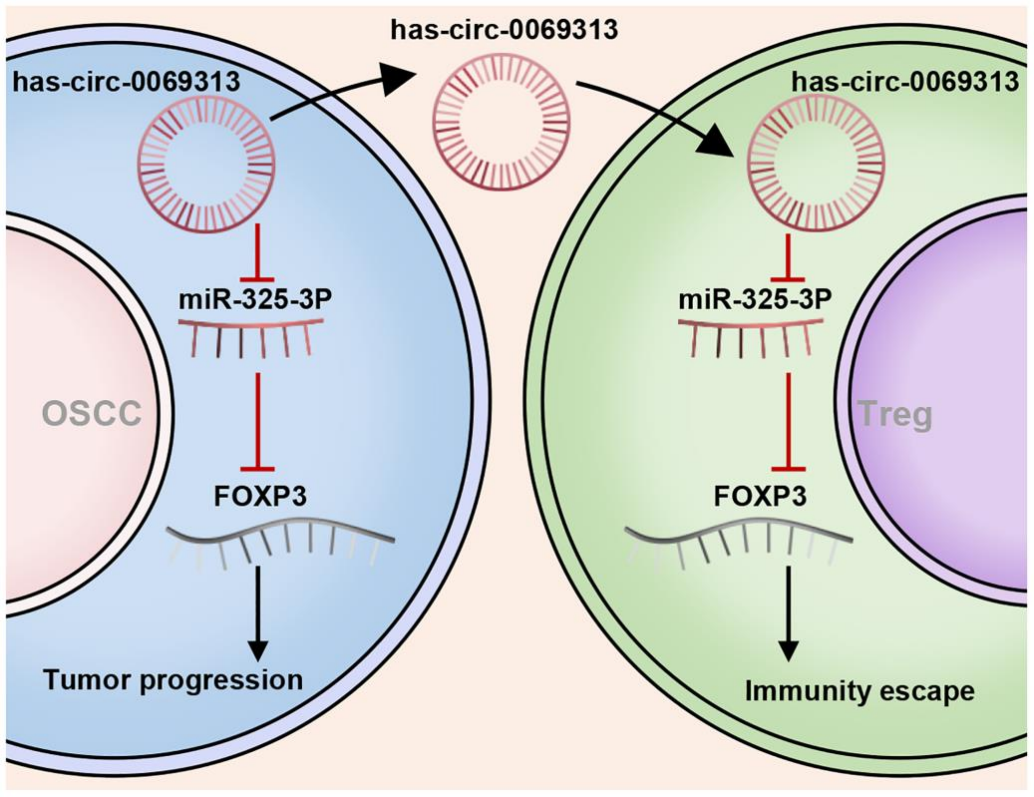
The schematical image of has_circ_0069313 on OSCC and Treg cells.

We applied a subcutaneous xenografts assay in C57BL/6n mice. Tumors were collected and tumor volume was detected (Figure 7A, 7B, ^***^p < 0.001). IHC was then applied to detected PDL1 and CD25 levels to measure the immunity status. The results showed that has_circ_0069313 knockdown cells developed impaired tumor growth and Treg cell infiltration. PDL1 and CD25 also decreased in has_circ_0069313 knockdown cells but increased in has_circ_0069313 overexpressing cells (Figure 7C, 7D, ^***^p < 0.001). taken together, Our results indicate that has_circ_0069313 induces OSCC immunity escape via the miR-325-3p-Foxp3 axis in both OSCC cells and Treg cells (Figure 8).

## DISCUSSION

CircRNAs are considered non-coding RNAs with mostly multi-functions in tumorigenesis and tumor progression [11]. Among these roles, miRNA sponge and competing endogenous RNAs represent the majority [12]. However, miRNA sponging circRNAs have various functions in different cancers because the regulated miRNAs and miRNA target mRNAs vary [13]. The second commonly accepted function is RNA binding protein partners. For example, circRNA-SORE mediates sorafenib resistance in hepatocellular carcinoma by targeting YBX1 [14]. CircRNAs promote the basal function of their target proteins that thus exert their biological functions. Specific circRNAs harbor dual functions in combinations. CircRNAs play key roles in the progression of OSCC and serve as biomarkers predicting the prognosis [15, 16]. However, the bio function and underlying mechanism of circRNAs in OSCC associated tumor immunity are largely unknown.

PDL1 is an important immunity checkpoint and is responsible for effector T cell inhibition [17]. Targeting PDL1/PD1 is the hottest tumor immunity therapy strategy and was effective in many kinds of cancers, including OSCCs [18]. However, targeting PDL1/PD1 is not efficient for all kinds of cancer, and effector T cell infiltration status or exhaust T cell status affect the immunity therapy efficiency [19]. Treg cells play key roles in innate immunity to keep the immunity reaction under control. In tumor-associated immunity therapy, Tregs inhibit the infiltration of effector T cells and promote cancer cell survival [20]. Cancer cells develop anti PDL1/PD1 resistance and targeting PDL1/PD1 may inhibit the basic function of Tregs causing side effects such as auto-immune diseases. Thus, targeting PDL1/PD1 doesn’t meet every demand, and understanding the regulating mechanism of PDL1 is urgently needed.

In this study, we detect the expression pattern of has_circ_0069313 in paired cancer and normal tissues. has_circ_0069313 was upregulated in cancer tissues and negatively correlated with the prognosis. We next established stable cell lines overexpression has_circ_0069313 and examined its function. has_circ_0069313 increases PDL1 levels in OSCC through sponging miR-325-3p and promoting PDL1 expression. has_circ_0069313 is an exosome circRNA that can paracrinally regulate Treg cells and promote Treg cell function. Taken together, these results indicate that has_circ_0069313 may be a potential therapeutic target for OSCC.

## METHODS

### Clinical sample collection and patient information

A total of 50 paired OSCC samples and normal adjacent tissues from patients who underwent surgical resection at the Hospital of Stomatology of Sun Yat-sen University, Guangzhou, between January 2015 and December 2016 were randomly collected. All OSCC tissue samples were diagnosed by pathology. All procedures were approved by the Ethics Committee of Guangzhou Women and Children’s Medical Center (202018100). Written and informed consent was obtained from patients. This study was conducted in accordance with the Declaration of Helsinki, the information of patients was shown in Table 1.

**Table 1:** Clinicopathological features in OSCC patients.

**Features**		**Case**
Age	≥60	22
	<60	28
Gender	Male	38
	Female	12
T classification	T_1_	20
	T_2_	22
	T_3_	6
	T_4_	2
LN metastases	Negative	34
	Positive	16
Clinical stage	C_I_	17
	C_II_	14
	C_III_	10
	C_IV_	9
Differentiation	Well	24
	Moderate	18
	Poor	8

Clinical stage was determined according to the 7th edition of the UICC-AJCC TNM staging system.

### Cell culture and transfection

Human immortalized HaCaT and oral SCC Cal-27 cells were maintained in DMEM (Sigma) medium supplemented with 10% fetal bovine serum and 1% penicillin-streptomycin, while other cell lines were maintained in complete DMEM-F12 medium. PcDNA3.1 vector containing has_circ_0069313 cDNA (Genechem, Shanghai, China) were transfected into OSCC cells with a low has_circ_0069313 level using Lipofectamine 3000 (Invitrogen, USA). All treated cells were selected with G418 (Millipore, MA, USA). Lentiviruses containing has_circ_0069313-targeting shRNAs (Genechem) were used to stably infect OSCC cells, which have a high has_circ_0069313 level. The sequences of shRNAs were as following, NC: TTGGCGCGTATGCAAC, ShRNA-1: CTTGTACATGCAATTGCGCGG, ShRNA-2: AAACTTGTACATGCAATTGCG.

### Western blot analysis

Equal protein was run on SDS-PAGE gel and transferred onto PVDF membranes. After blocking, the membranes were then incubated with primary antibodies overnight at 4°C, washed with TBST three times, and then incubated with appropriate secondary antibodies for 1 h at room temperature. Target proteins were detected with ECL (Millipore) reagent. The antibodies involved in the manuscript were as below: PDL1 (Cell Signaling Technology, cat# 13684), β-actin (CST, cat# 3700), CD8 (CST, cat# 85336), CD25 (CST, cat# 39475), Foxp3 (CST, cat# 12653), GM130 (CST, cat# 12480), CD54 (CST, cat# 67836), CD9 (CST, cat# 98327).

### Immunohistochemistry (IHC)

Briefly, paraffin-embedded tissues were cut at a 6–10 μM thickness and were deparaffinized in xylene and then rehydrated. Antigens were restored and blocked with goat serum dilution buffer for 1 h at room temperature. The tumor sections were incubated with primary antibodies in a wet chamber overnight at 4°C and then secondary antibodies were added for incubation at room temperature for 1 h. The tumor sections were subsequently visualized by Diaminobenzidine (DAB) reagent and then counterstained with hematoxylin for detection. Representative images were from at least three independent experiments.

### RNA fluorescence *in situ* hybridization (FISH)

The coverslips seeded with cells were incubated in the incubator and fluorescently labeled junction probe for 12 hours. Then the cells were rinsed 3 times and incubated at room temperature overnight. Images were taken using Confocal microscopy and representative images were then picked. The sequence of the circRNA detection probe was as follows: 5′ cy3-TAGAAGCCTGGACCTTCTTGGG 3′.

### Reverse transcription and real-time (RT) PCR

Total RNA was isolated with a PureLink RNA mini kit (Thermo Fisher Scientific, MA, USA) according to manufacturer’s instruction. RNA was reverse-transcribed into cDNA and then subjected to RT–PCR analysis with SYBR Select Master Mix (Thermo Fisher Scientific) in a StepOne Plus real-time PCR system (Applied Biosystems). β-actin was used as internal control. The key primers are listed below: has_circ_0069313-F: CCAGAGGACAGTTCCTGGAC; has_circ_0069313-R: AGATGGCATGAGGGATATCG.

### Dual luciferase activity reporter system

The Renilla luciferase (Rluc) and firefly luciferase (Luc) sequences were cloned into the reporting plasmid, The Foxp3 sequence along with its 3′UTR was amplified and inserted between Rluc and Luc. Relative activity was calculated by determining the ration of Rluc/Luc.

### RNA immunoprecipitation assay

Magna RIP Kit (Millipore) was used for RNA immunoprecipitation (RIP) assay. Briefly, the cells were lysed with RIP lysis buffer and then incubated with magnetic beads. Afterwards, proteinase K was added for purification of RNA. The enriched RNA was analyzed by qRT–PCR further analysis.

### OSCC derivation exosome collection

Exosomes derived from OSCC were performed through differential ultracentrifugation. Briefly, cells cultures were centrifuged at 4°C to obtain supernatant and then centrifuged at 10,000 × g for 20 min, 70 min and 60 min respectively.

### T cell isolation

Human Treg (CD4+ CD25hi) were purified from PBMCs from healthy donors, after staining with the following antibodies at 1:100 dilution: FITC anti-human CD4 (BD PharMingen, clone RPA-T4, Cat.# 555346), PE anti-human CD25 (BD PharMingen, clone M-A251, Cat.# 555432). Treg cells were treated with Detach reagent (Invitrogen) to remove antibody. Treg cells were thus harvested and subjected to the following experiments.

### Animal studies

The C57BL6/n nude mice (female, 4 weeks old) were obtained from the Laboratory Animal Center of Sun Yat-sen University (L102012020086). All animals were maintained under the guidance of the Committee on Animals of Sun Yat-sen University. A total of 5 × 10^6^ cells were subcutaneously injected into the right flanks for establishment of xenograft model. The tumor samples were harvested and further subjected to IHC staining. Representative images were from at least three independent experiments.

### Statistical analysis

Statistical analyses were analyzed using SPSS 20.0 statistical version. The significance between two groups were compared by Student’s *t*-test. OS was analyzed by Kaplan-Meier methods, and P less than 0.05 was considered to be a statistically significant difference from the control.

### Availability of data and material

The datasets used and/or analyzed during the current study are available from the corresponding author on reasonable request.
